# “The Organon” in Homœopathic Medical Colleges

**Published:** 1885-01

**Authors:** 


### "THE ORGANON" IN HOMŒOPATHIC MEDICAL COLLEGES.

A graduate of last spring of one of our colleges, not "one of the best," recently when questioned as to acquaintance with the contents of the *Organon*, had never seen the book; had heard there was such a book, but as to its contents or purposes had never heard, and as to these confessed utter ignorance. Yet this graduate had the certificate of this college to fitness and ability to practice in this community a system of practical medicine the philosophy of which had never been seen or heard of. Is any condemnation too sharp for neglect and knavery like this? Can we but say—How long shall our patience be abused?

We receive with great pleasure and thankfulness the statement that there is *one* college in the country where the *Organon* is taught, and that this is found in St. Louis. Is there another? If there be, its whereabouts is unknown.—P. P. Wells.

The above extract does great injustice to at least two, and probably more than two, of our prominent medical colleges. St. Louis is not entitled to the honor of being the only city where the *Organon* of Hahnemann is respected and taught in its medical schools. Professor Mitchell, when shown the above quotation, assured us that there had not been a year since the organization of the Chicago Homœopathic Medical College when the *Organon* had not been the special subject of *one or more* lectures, and that references thereto are frequently made. The same can be said of the Hahnemann Medical College of this city.—*Medical Era.*

"One or more lectures!" "References thereto are frequently made!!" Are these few lectures and occasional references sufficient to teach thoroughly the philosophy of Homœopathy? Not much! If this be all the instruction given upon the *Organon* in "our prominent colleges," we fail to see any injustice to "our prominent colleges" in Dr. Wells' article.

